# Comment on “significant decrease in plasma d‐dimer levels and mean platelet volume after a 3‐month treatment with rosuvastatin in patients with venous thromboembolism”

**DOI:** 10.1002/clc.23917

**Published:** 2022-09-08

**Authors:** Erkan Coban

**Affiliations:** ^1^ Department of Internal Medicine, Faculty of Medicine Akdeniz University Antalya Turkey

To the Editor,

1

I read the research study conducted by Alirezaei et al.^1^ related with “significant decrease in plasma d‐dimer levels and mean platelet volume (MPV) after a 3‐month treatment with rosuvastatin in patients with venous thromboembolism” which was published in your journal, with great interest. Authors reported that a 3‐month treatment with 10 mg rosuvastatin daily can significantly decrease the plasma levels of d‐dimer and MPV, which would support a potential role of statins to reduce activated systemic inflammation among venous thromboembolism patients. I would like to emphasize the existence of some factors that may adversely affect the MPV data in this study.

In this study, MPV measurement technique is not written. Preanalytical variables, such as the anticoagulant used, and the time between blood collection and measurement are known to significantly affect MPV measurements. Although ethylenediaminetetraacetic acid (EDTA) is traditionally used and recommended for samples destined for blood counting it is well known that platelets collected into EDTA anticoagulants undergo time‐dependent platelet swelling and activation. MPV variability due to the use of EDTA as an anticoagulant has been reported between 2% and 50% in different studies.^2^, ^3^, ^4^
